# Recent developments in the asymmetric Reformatsky-type reaction

**DOI:** 10.3762/bjoc.14.21

**Published:** 2018-02-02

**Authors:** Hélène Pellissier

**Affiliations:** 1 Aix-Marseille Univ, CNRS, Centrale Marseille, iSm2, Marseille, France

**Keywords:** asymmetric (aza)-Reformatsky reaction, asymmetric synthesis, chirality, diastereoselectivity, enantioselectivity, total synthesis

## Abstract

This review collects the most important developments in asymmetric Reformatsky-type reactions published since the beginning of 2013, including both diastereoselective methodologies based on the use of chiral substrates and enantioselective catalytic procedures.

## Introduction

**Scheme 1:**
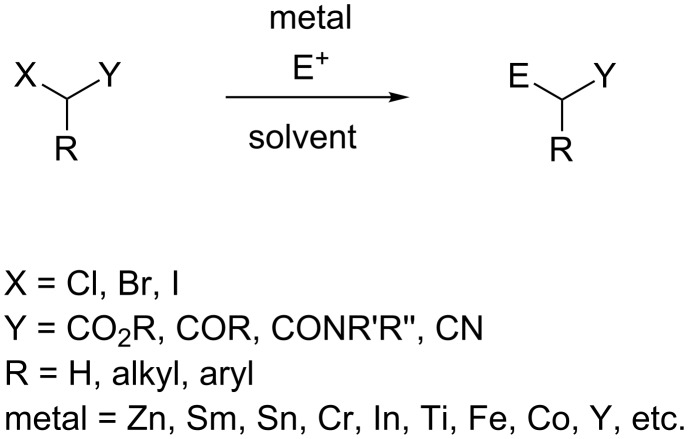
Reformatsky-type reaction.

The Reformatsky reaction involves the formation of β-hydroxyalkanoates from the reaction of α-halocarbonyl compounds with aldehydes or ketones in the presence of zinc [1]. Today, the Reformatsky reactions are more generally defined as metal insertion into carbon–halogen bonds which are activated by carbonyl groups or related functions at vicinal or vinylogous positions followed by condensation to electrophiles (Scheme 1). The Reformatsky reagent is usually in situ generated by the treatment of the corresponding α-halocarbonyl compound by a metal in low oxidation state (Zn, Sm, Sn, Cr, In, Ti, Fe, Co, Y, etc.).

These robust carbon–carbon bond-forming reactions constitute efficient alternatives to base-induced aldol condensations since the enolates are formed under neutral conditions, which allow an excellent functional group tolerance. However, the Reformatsky reaction generally provided lower yields and stereoselectivities along with narrower substrate scopes than the aldol reaction, especially in the case of heterogeneous conditions. Moreover, another limitation of the Reformatsky reaction is the requirement of an α-halogen in the Reformatsky reagent. Anyway, the Reformatsky reaction is versatile since it can occur through either intra- or intermolecular fashion leading to cyclic as well as acyclic products, and includes (aza)-versions using imines instead of aldehydes or ketones as electrophilic partners. In addition, the mild reaction conditions usually employed and the use of cheap nontoxic metals has prompted the development of diastereo- and enantioselective (aza)-variants by using chiral substrates or chiral ligands. As illustrated in this review, the diastereoselective Reformatsky reactions of chiral substrates or auxiliaries have been applied to the total synthesis of a number of important products, such as naturally occurring bioactive products. Furthermore, the challenging field of catalytic enantioselective (aza)-versions is fast-growing since the discovery that ZnMe_2_ or ZnEt_2_ could be used as zinc sources allowing more convenient homogeneous reaction conditions to be used [2–3]. This review collects the important developments in asymmetric Reformatsky-type reactions published since the beginning of 2013, since this field was most recently reviewed this year by Colobert for the diastereoselective version [4], and Fernández-Ibáñez and Maciá for the catalytic enantioselective version [5]. Earlier, this field was reviewed by different groups [6–13]. Moreover in 2014, two book chapters dealing with organozinc reagents were published covering the literature up to 2011 [14–15], and a review focusing on general SmI_2_-mediated cross-coupling reactions including only two references dated of 2013 concerning diastereoselective Reformatsky reactions [16]. The present review is divided into two parts, dealing successively with diastereoselective (aza)-Reformatsky reactions of chiral substrates or auxiliaries and catalytic enantioselective (aza)-Reformatsky reactions using chiral ligands.

## Review

### Diastereoselective Reformatsky reactions

#### Intermolecular Reformatsky reactions

**Scheme 2:**
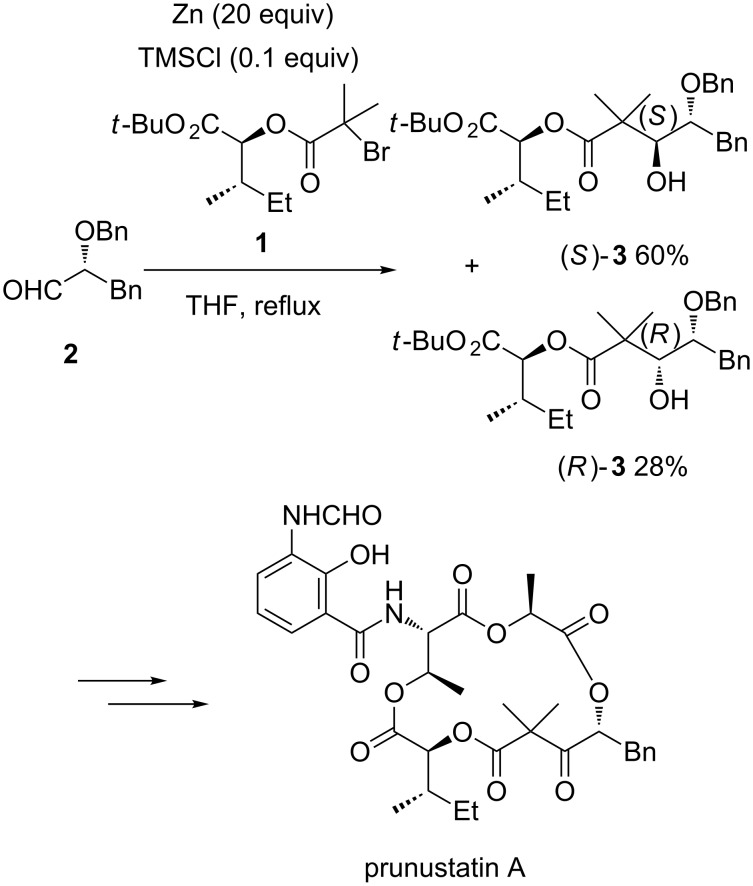
First total synthesis of prunustatin A based on a Zn-mediated Reformatsky reaction [17].

Chiral β-hydroxyalkanoates constitute key intermediates in the synthesis of biologically active molecules. Diastereoselective zinc-mediated Reformatsky reactions involving either chiral aldehydes or chiral nucleophiles or both of them have been widely applied to prepare these products. For example, this methodology was employed in 2014 by Kawanishi and Yamakoshi to develop the first total synthesis of naturally occurring prunustatin A, a novel inhibitor of glucose-regulated protein 78 expression [17]. As shown in Scheme 2, the key step of the synthesis was a zinc-mediated Reformatsky reaction between chiral bromide **1** and chiral aldehyde **2** performed in THF at reflux, leading to a 68:32 (36% de) mixture of the corresponding (*S*)*-* and (*R*)-alcohols **3** in 88% combined yield. The latter were subsequently converted through ten supplementary steps, including a 15-membered ring macrocyclization, into the expected prunustatin A.

**Scheme 3:**
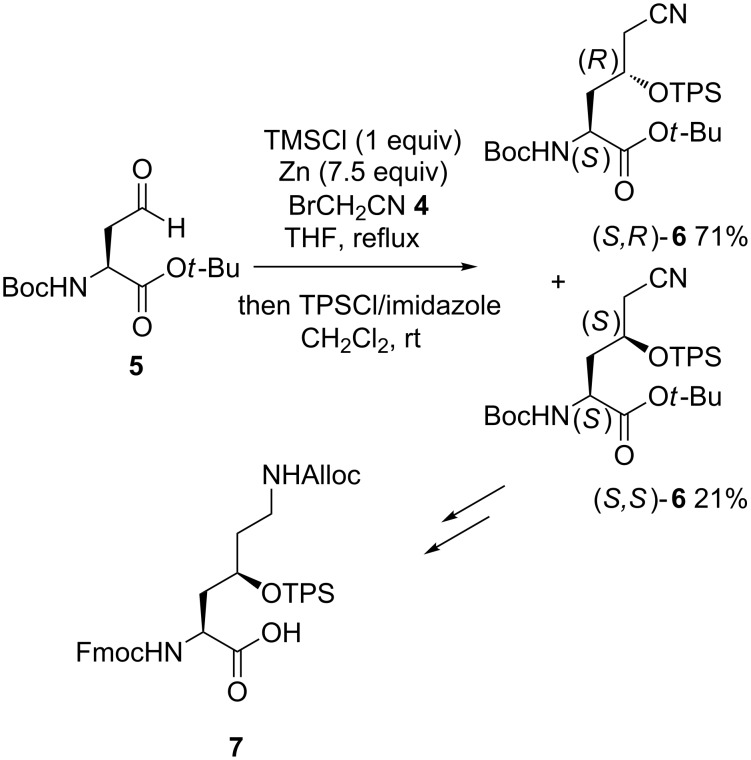
Synthesis of a γ-hydroxylysine derivative through a Zn-mediated nitrile Reformatsky-type reaction [18].

In 2015, a nitrile Reformatsky-type reagent in situ generated from bromoacetonitrile (**4**) and zinc in the presence of TMSCl was added to chiral aldehyde **5** derived from L-aspartic acid [18]. The reaction, performed in THF at reflux followed by protection of the formed alcohols as silyl ethers by treatment with TPSCl (triphenylsilyl chloride), provided the corresponding chiral protected Reformatsky products **6** in 92% yield as a 77:23 mixture of (*S*,*R*)- and (*S*,*S*)-diastereomers (Scheme 3). In spite of its moderate diastereoselectivity (54% de), the utility of this procedure was demonstrated by converting the minor (*S*,*S*)-diastereomer **6** into the orthogonally protected γ-hydroxylysine derivative **7** which is found in the potent antitumor agent glidobactin A [19].

**Scheme 4:**
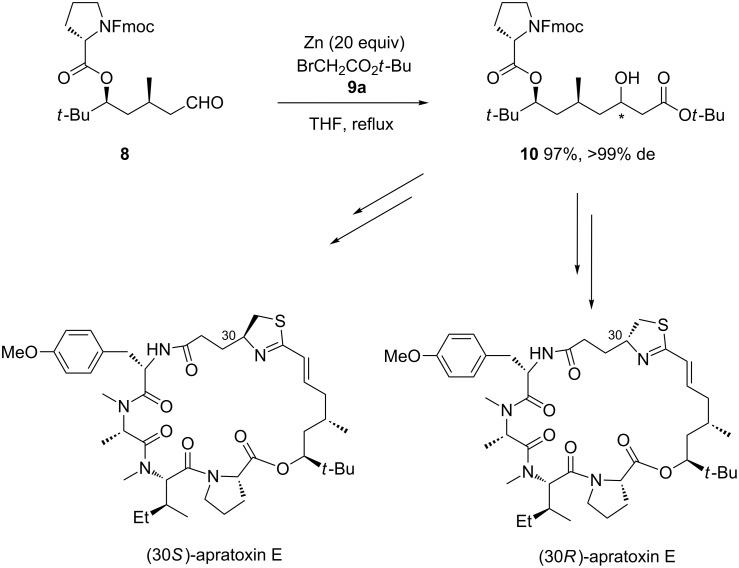
Synthesis of apratoxin E and its C30 epimer through a Zn-mediated Reformatsky reaction. Fmoc = 9-fluorenylmethoxylcarbonyl [20].

Another chiral electrophile, such as aldehyde **8** prepared in five steps from (−)-citronellal, was submitted by Luesch and Zhang to diastereoselective zinc-mediated Reformatsky reaction with *tert-*butyl bromoacetate (**9a**) in THF at reflux [20]. The process afforded in almost quantitative yield (97%) the corresponding β-hydroxy ester **10** as a single diastereomer, as illustrated in Scheme 4. This constituted the key step of the first total synthesis of (30*S*)-apratoxin E and its (30*R*)-epimer. Comparing the spectroscopic data of these two diastereomers with those of natural apratoxin E unambiguously confirmed that (30*R*)-apratoxin E was the natural product and that (30*S*)-apratoxin E was actually 30-epiapratoxin E, thus correcting the originally proposed absolute configuration. Both diastereomers were tested for their antiproliferative activities in HCT116 cells, showing that natural (30*R*)-apratoxin E exhibited a higher activity than its (30*S*)-epimer.

**Scheme 5:**
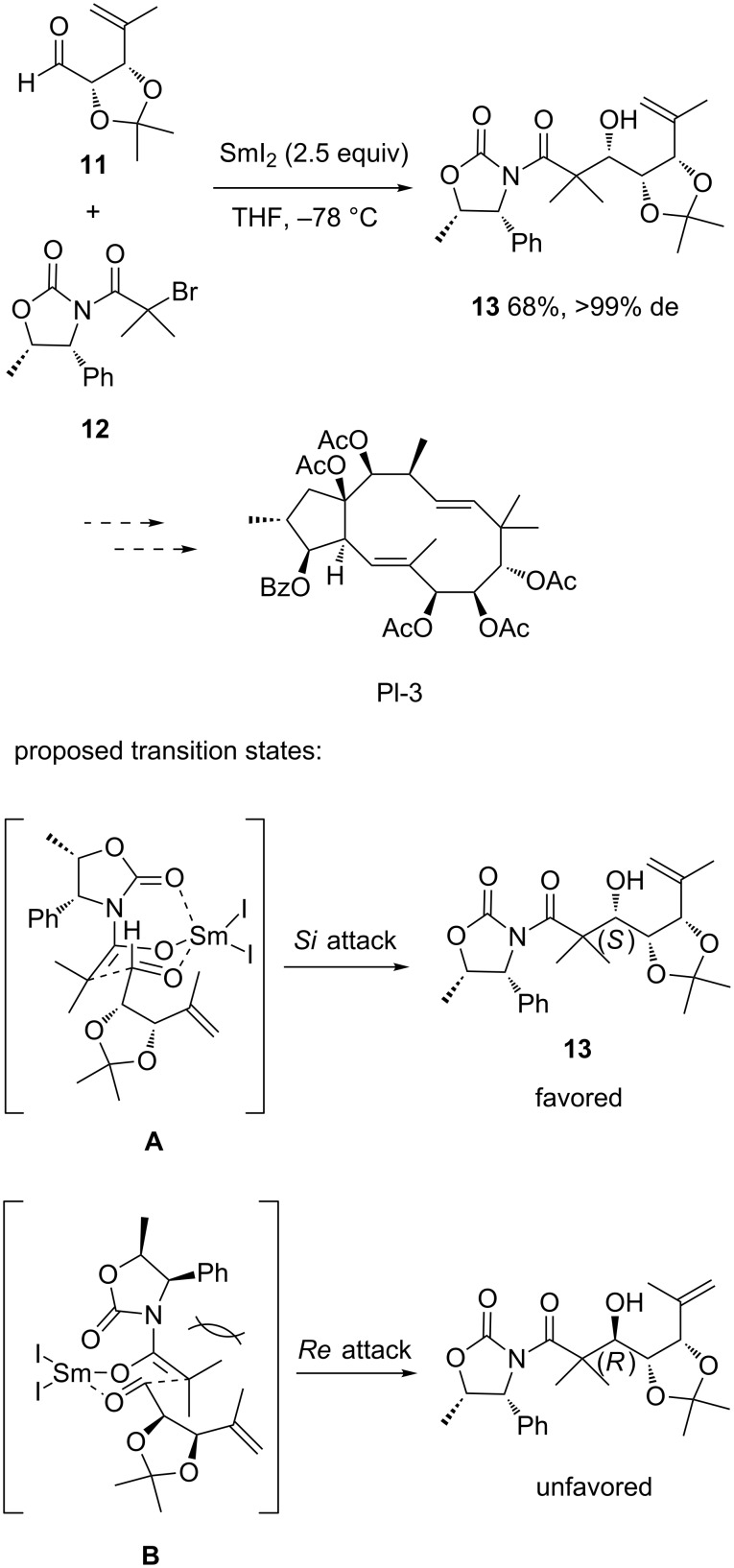
Synthesis of the eastern fragment of jatrophane diterpene Pl-3 through a SmI_2_-mediated Reformatsky reaction [21].

Due to their lower basicity, samarium enolates can be considered as valuable alternatives to lithium enolates. Consequently, a number of samarium-mediated Reformatsky reactions have been developed. For example, SmI_2_ was found by Rinner et al. to mediate the diastereoselective Reformatsky reaction between chiral aldehyde **11** derived from D-ribonolactone and chiral bromide **12** prepared from D-ribose [21]. On the basis of this double induction, the Reformatsky reaction performed at −78 °C in THF provided a complete diastereoselectivity, affording the corresponding chiral product **13** as a single diastereomer in 68% yield (Scheme 5). This process constituted the key step of a nine-step total synthesis of the eastern fragment of jatrophane diterpene Pl-3, which is a complex natural product with high promising biological activities, such as antiproliferative activity and inhibition of the efflux-pump activity of multidrug resistance P-glycoprotein. The high stereoselectivity of the process could be explained by to the exclusive formation of the pentacoordinated transition states **A** and **B** (Scheme 5). Indeed, steric repulsion between the auxiliary and the bulky dimethyl group prevented a *Re* attack through transition state **B**, thus forcing the system to undergo a *Si* attack through transition state **A**.

**Scheme 6:**
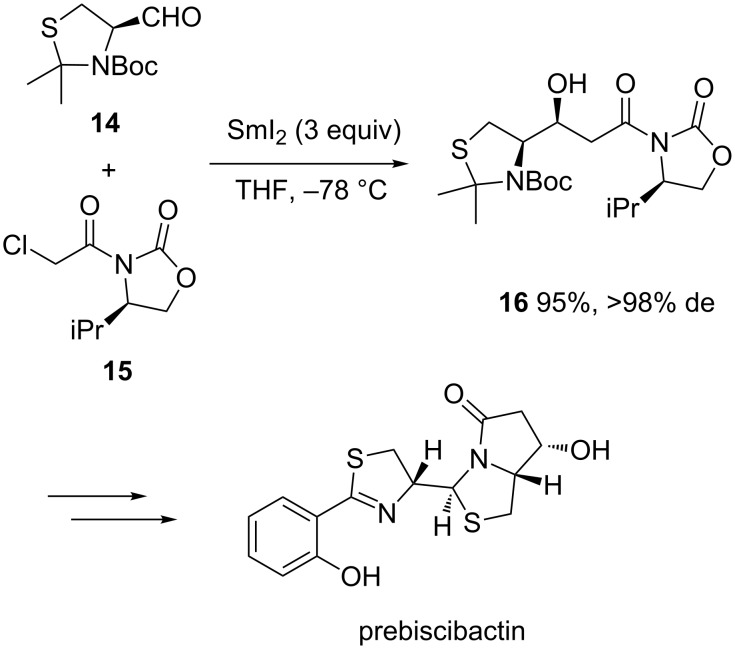
First total synthesis of prebiscibactin through a SmI_2_-mediated Reformatsky reaction. Boc = *tert*-butoxycarbonyl [22].

A related methodology was also applied by Jiménez and Rodriguez to develop the first total synthesis of prepiscibactin, which is an intermediate in the biosynthesis of piscibactin [22]. Indeed, the sequence was based on the diastereoselective SmI_2_-mediated Reformatsky reaction between L-cysteine-derived thiazolidinic aldehyde **14** and (*R*)-α-chloroacetyl-2-oxazolidinone **15**, leading to the corresponding β-hydroxy amide **16** in both remarkable yield (95%) and diastereoselectivity (>98% de), as shown in Scheme 6. The latter was subsequently converted into the expected prepiscibactin through five supplementary steps, including Zn(II)-induced thiazolidine formation followed by lactamization. On the basis of NOE methods, the synthetic prebiscibactin was found spectroscopically indistinguishable from the natural product.

**Scheme 7:**
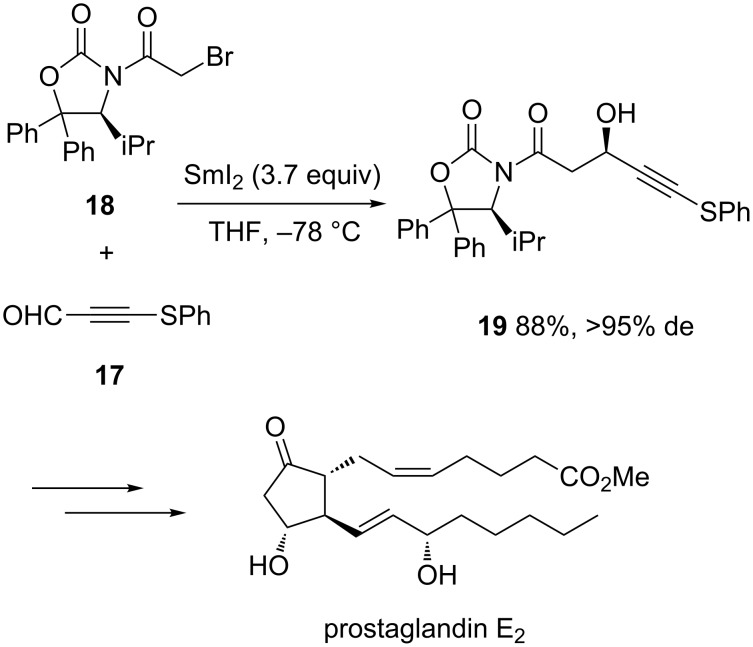
Synthesis of prostaglandin E_2_ methyl ester through a SmI_2_-mediated Reformatsky reaction [23].

More recently, excellent results were also reported by Isobe et al. for a diastereoselective SmI_2_-mediated Reformatsky reaction constituting the key step of a highly economical total synthesis of prostaglandin E_2_ methyl ester [23]. As shown in Scheme 7, the reaction occurred between achiral aldehyde **17** and chiral oxazolidinone **18** in the presence of SmI_2_ in THF at −78 °C to give the corresponding chiral secondary alcohol **19** in high yield (88%) combined with excellent diastereoselectivity (>95% de). Six supplementary steps allowed prostaglandin E_2_ methyl ester to be achieved in 24% overall yield.

**Scheme 8:**
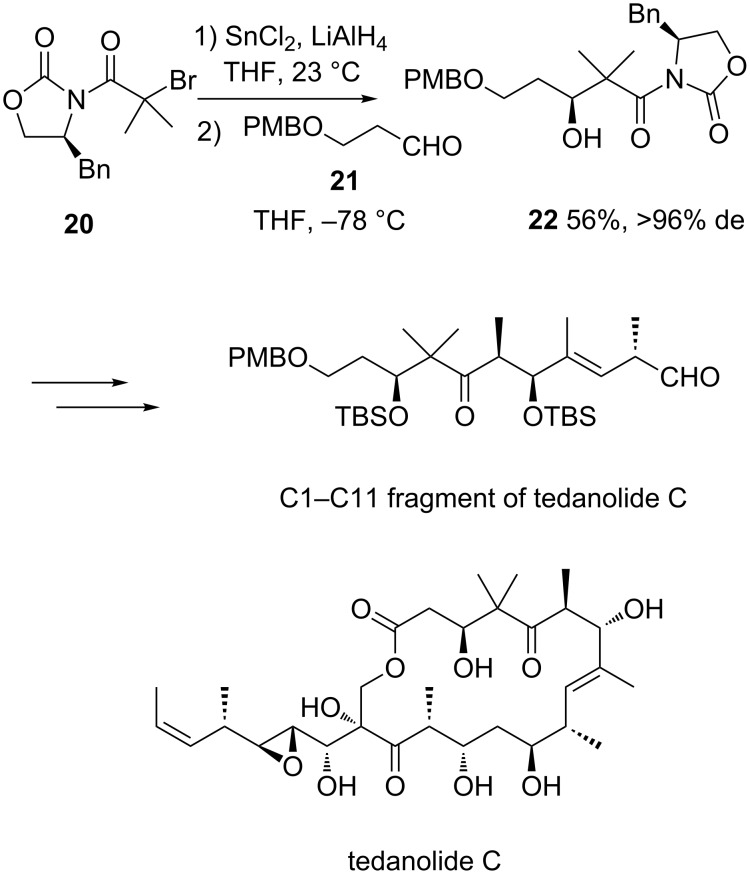
Synthesis of the C1–C11 fragment of tedanolide C through a SnCl_2_-mediated Reformatsky reaction. PMB = *p*-methoxybenzyl. TBS = *tert*-butyldimethylsilyl [24].

In addition to zinc and samarium, SnCl_2_ is also capable to mediate Reformatsky reactions. As an example, chiral oxazolidinone **20** reacted with achiral aldehyde **21** in the presence of SnCl_2_ to afford the corresponding chiral Reformatsky product **22** as a single diastereomer (>96% de) in moderate yield (56%), as shown in Scheme 8 [24]. The latter was used to synthesize the C1–C11 fragment of naturally occurring protein inhibitor tedanolide C.

#### Intermolecular aza-Reformatsky reactions

**Scheme 9:**
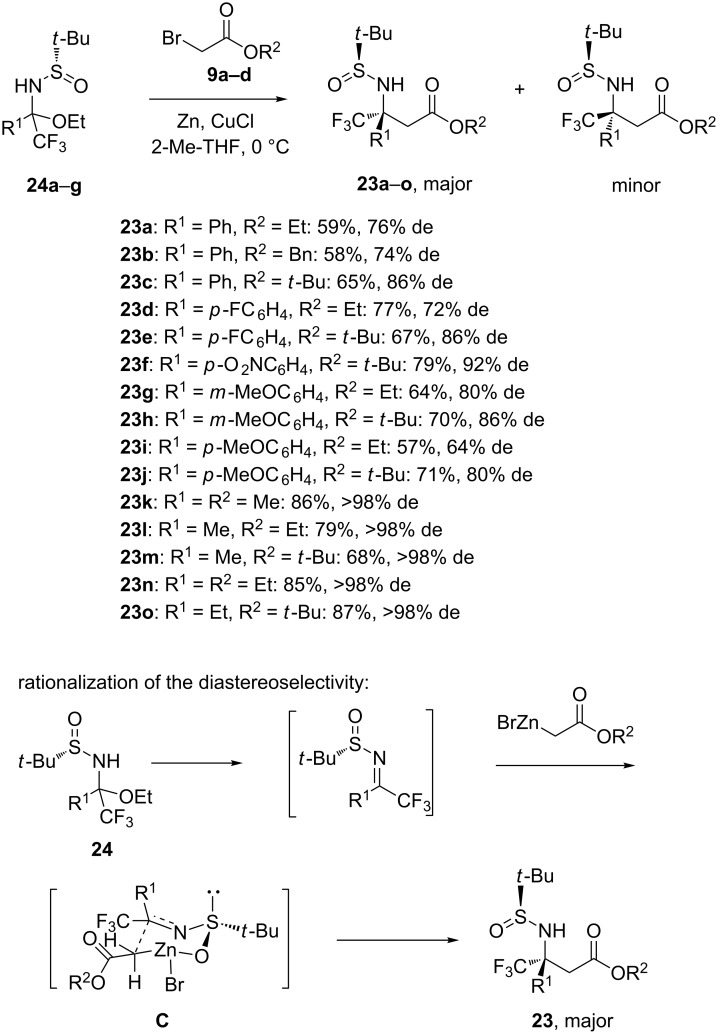
Synthesis of β-trifluoromethyl β-(*N*-*tert-*butylsulfinyl)amino esters exhibiting a quaternary stereocenter through a Zn-mediated aza-Reformatsky reaction [26].

The diastereoselective zinc-mediated aza-Reformatsky reaction represents a powerful and direct methodology to prepare chiral β-aminoesters [25], which represent key intermediates in total synthesis. In 2013, Grellepois described the synthesis of chiral β-aryl(alkyl) β-trifluoromethyl β-amino esters **23a–o** based on the highly diastereoselective zinc-mediated addition of Reformatsky reagents, in situ generated by treatment of bromoesters **9a**–**d** by Zn in the presence of CuCl in 2-Me-THF, to chiral α-aryl(alkyl) α-trifluoromethyl *N-tert-*butylsulfinyl hemiaminals **24a**–**g**, constituting bench-stable surrogates of trifluoromethyl ketoimines [26]. As illustrated in Scheme 9, a range of these chiral aza-Reformatsky products **23a**–**o** were obtained in moderate to high yields (57–87%) combined with moderate to excellent diastereoselectivities (64–98% de). The best diastereoselectivity (>98% de) was achieved in the formation of β-alkyl β-trifluoromethyl β-amino esters **23k**–**o** (R^1^ = Me or Et) while moderate to high diastereoselectivities (64–92% de) were obtained for the synthesis of β-aryl β-trifluoromethyl β-amino esters **23a**–**j**. While the diastereoselectivities were found sensible to the size of the alkyl bromoacetate used in the reaction of aryl-substituted substrates, single diastereomers (>98% de) were obtained from alkyl-substituted substrates regardless of the nature of the bromoacetate employed. The stereochemistry of aza-Reformatsky reactions involving *N*-sulfinyl ketimines is generally explained through a six-membered transition state with the zinc coordinated to the sulfinyl oxygen atom and the enolate carbanion to the imino carbon atom. The other substituents accommodate in order to minimize the steric repulsions in this transition state. Therefore in transition state **C** (Scheme 9), the trifluoromethyl group occupied the equatorial position instead of the axial position because of sterical hindrance and electrostatic repulsion between the lone pair of the sulfur atom and the trifluoromethyl group. It must be noted that this methodology represented the first efficient and general preparation of chiral β-trifluoromethyl β-amino acid derivatives containing a quaternary stereocenter adjacent to the amine function.

**Scheme 10:**
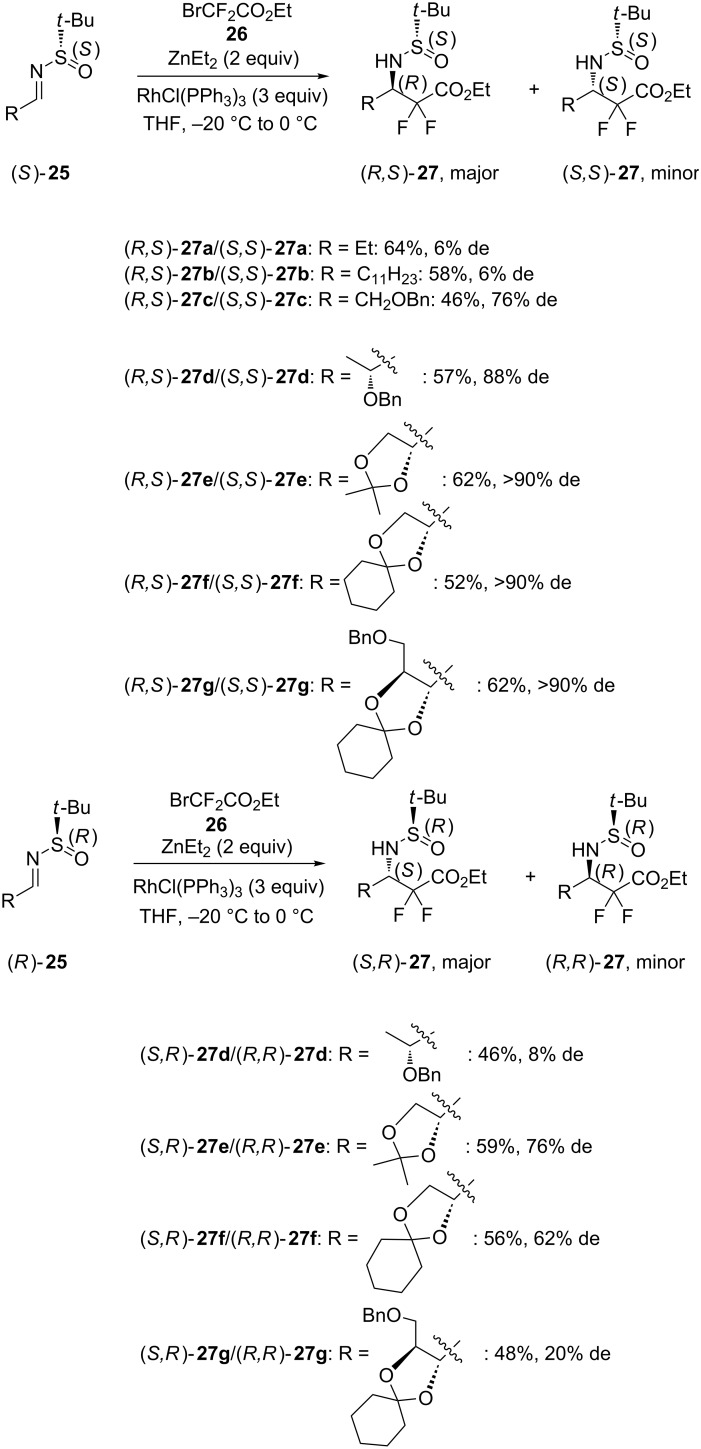
Synthesis of α,α-difluoro-β-(*N*-*tert-*butylsulfinyl)amino esters through Zn(II)-mediated aza-Reformatsky reactions [27].

In 2014, Linclau and Poisson reported the synthesis of chiral α,α-difluoro-β-(*N*-*tert-*butylsulfinyl) amino esters through ZnEt_2_-mediated aza-Reformatsky reactions of the corresponding chiral α-oxygenated sulfinyl imines **25** with ethyl bromodifluoroacetate (**26**) performed in the presence of RhCl(PPh_3_)_3_ as an additive in THF [27]. Starting from (*S*)-*N*-*tert-*butylsulfinyl imines **25**, the reaction led to the corresponding (*R*,*S*)-products **27** as major diastereomers in low to excellent diastereoselectivities (6–90% de) and moderate yields (46–64%). The lowest diastereoselectivity (6% de) was obtained in the reaction of aliphatic sulfinyl imines (*S*)-**25a**,**b** (R = Et, C_11_H_23_) while that of sulfinyl imines (*S*)-**25c**,**d** exhibiting an α-benzyloxy substituent provided high diastereoselectivities (76–88% de). This assumed the coordination of the α-alkoxy group to zinc. Even a higher diastereoselectivity of >90% de was achieved for more sterically hindered α-alkoxy-derived substrates (*S*)-**25e**–**g** (Scheme 10). When the enantiomeric (*R*)-*N*-*tert-*butylsulfinyl imines **25d**–**g** were employed as substrates, the diastereoselectivity of the reaction was much reduced, leading to the corresponding (*S,R*)-products **27d**–**g** in low to good diastereoselectivities (8–76% de). Moreover, products (*S,R*)-**27d**–**g** were obtained in slightly lower yields (46–59% vs 52–62%) than the corresponding (*R,S*)-products **27d**–**g**, as shown in Scheme 10.

**Scheme 11:**
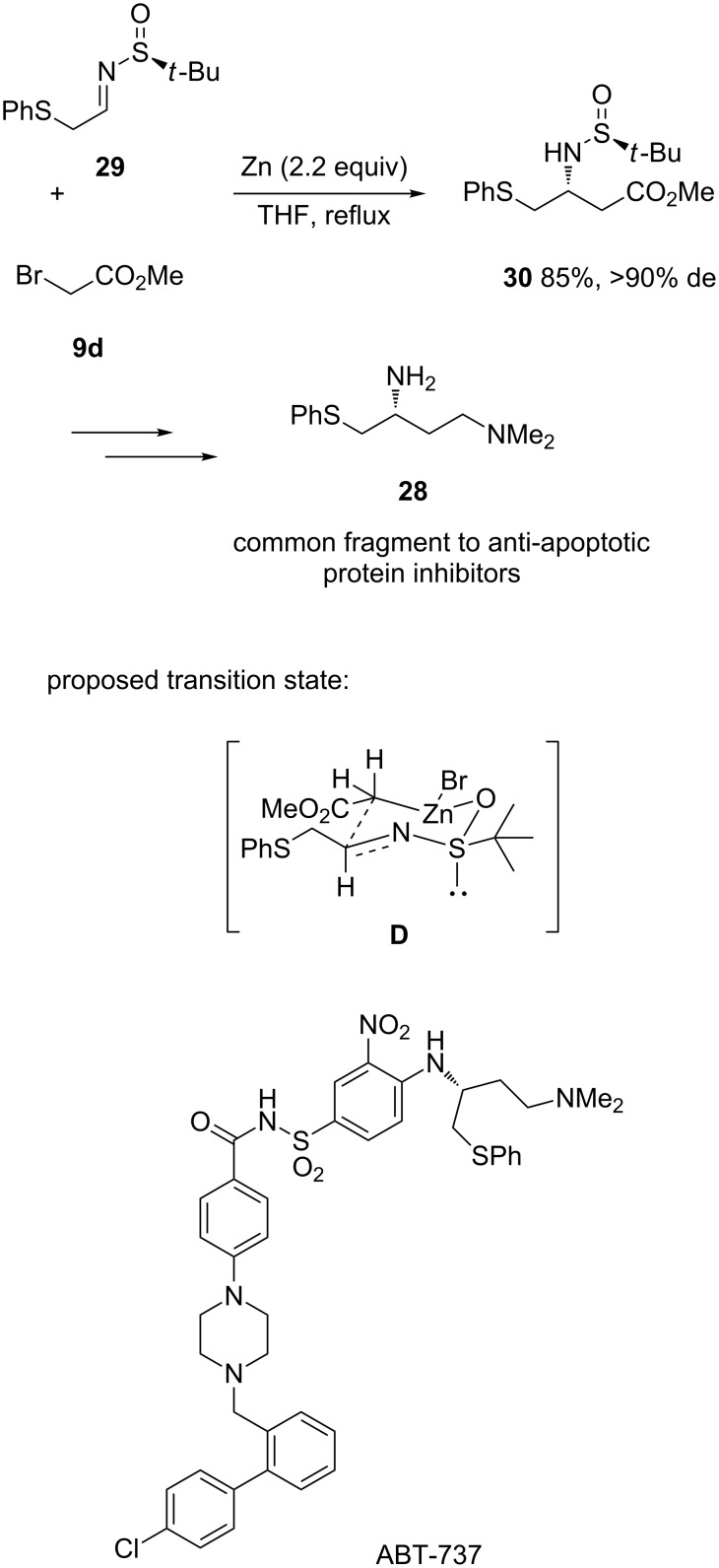
Synthesis of a common fragment to anti-apoptotic protein inhibitors through a Zn-mediated aza-Reformatsky reaction [28].

A related methodology was used by Levacher and Hardouin to develop a novel synthesis of primary chiral diamine **28**, which is a common fragment to anti-apoptotic protein inhibitors, such as ABT-737 [28]. As shown in Scheme 11, the key step of this synthesis was a highly diastereoselective zinc-mediated aza-Reformatsky reaction of methyl bromoacetate (**9d**) with enantiopure *N*-*tert-*butylsulfinyl imine **29**. It led to the corresponding chiral β-amino ester **30** in both high yield (85%) and diastereoselectivity (>90% de), as shown in Scheme 11. To explain the stereoselectivity of the process, the authors have proposed the six-membered transition state **D** (Scheme 11). Product **30** was further converted into expected chiral diamine **28** through three supplementary steps.

**Scheme 12:**
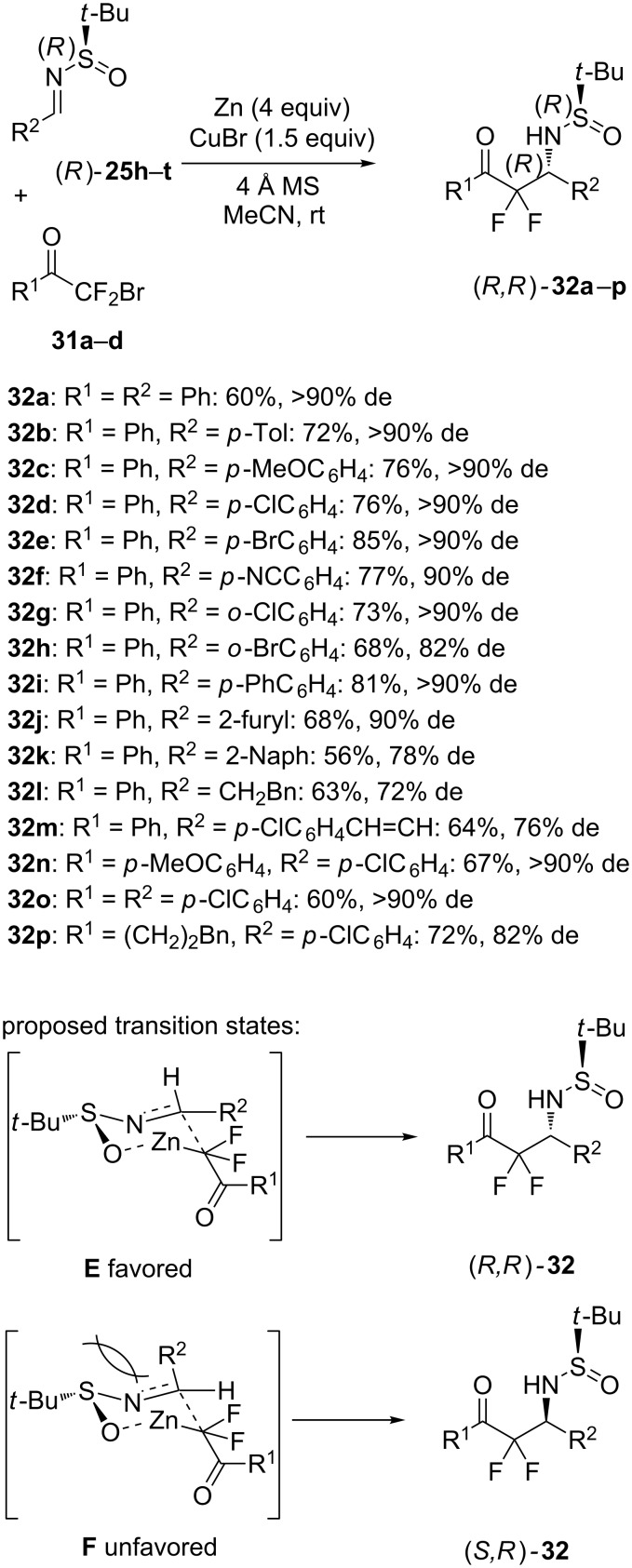
Synthesis of α,α-difluoro-β-(*N*-*tert-*butylsulfinyl)amino ketones through a Zn-mediated aza-Reformatsky reaction [29].

Later in 2015, Liu et al. developed Zn-mediated aza-Reformatsky reactions of chiral *N*-*tert-*butylsulfinyl imines (*R*)-**25h**–**t** with bromodifluoromethyl ketones **31a–d** in the presence of CuBr [29]. The reactions were performed in acetonitrile at room temperature, leading to the corresponding chiral α,α-difluoro-β-(*N*-*tert-*butylsulfinyl)amino ketones (*R*,*R*)-**32a**–**p** in moderate to good yields (56–85%) and moderate to excellent diastereoselectivities (72–90% de). As illustrated in Scheme 12, the asymmetric process tolerated a broad range of functional groups. For example, the reactions of chiral ketimines **25i**–**o** bearing methyl, methoxy, halides, and cyano substituents all afforded the corresponding chiral products **32b**–**h** in good yields (68–85%) and good to high diastereoselectivities (82–90% de). Imines derived from 4-biphenylcarbaldehyde or furfural also reacted smoothly, giving chiral products **32i**,**j** with excellent diastereoselectivities (≥90% de). However, slightly lower diastereoselectivities (72–78% de) were obtained for chiral products **32k**–**m** arisen from the corresponding naphthyl, alkyl or vinyl-substituted imines. Concerning the scope of the ketone partner, in addition to bromodifluoromethyl phenyl ketone (**31a**), *p*-methoxy- and *p*-chloro-substituted phenyl ketones **31b**–**c** provided moderate yields (60–67%) and excellent diastereoselectivity (>90% de). Moreover, an aliphatic ketone **31d** (R^1^ = (CH_2_)_2_Bn) was compatible to the reaction conditions, affording the corresponding product **32p** in 72% yield and 82% de. To explain these results, the authors have proposed the six-membered transition state **E** affording (*R,R*)-**32** as major product. Indeed, transition state **F** is unfavored due to repulsive steric interactions between the chiral auxiliary and the ketamine R^2^ group.

**Scheme 13:**
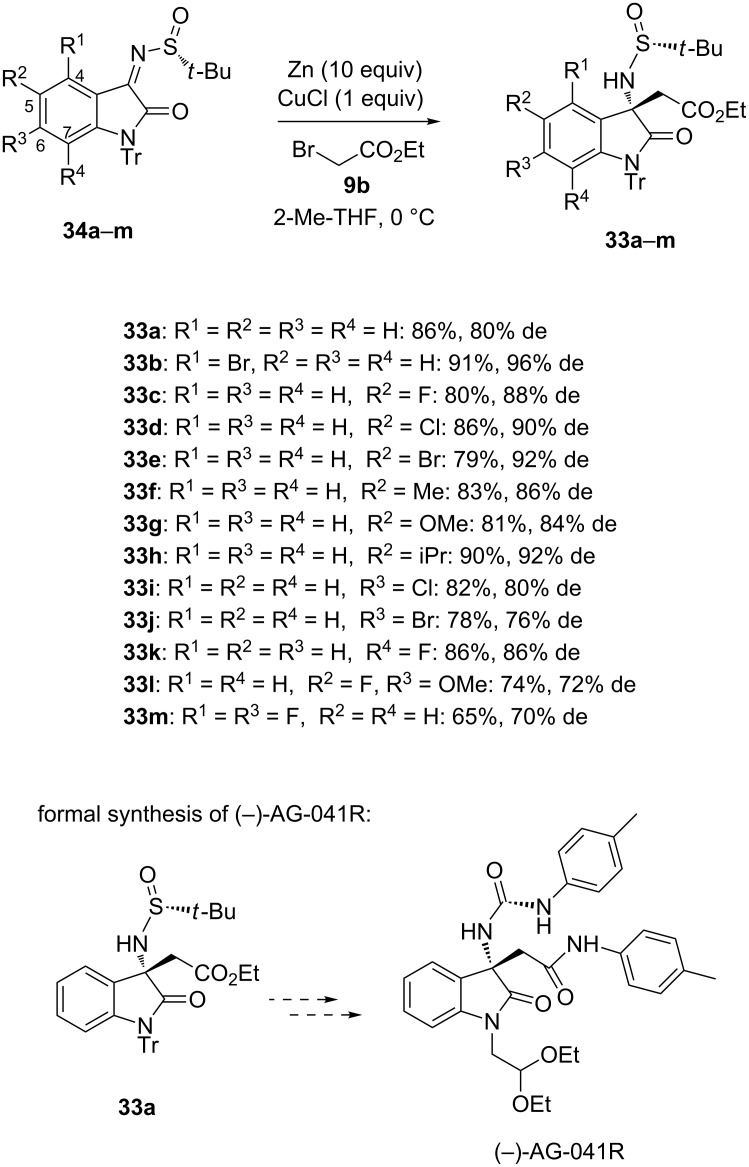
Synthesis of (2-oxoindolin-3-yl)amino esters through a Zn-mediated aza-Reformatsky reaction [30].

In 2016, Xu and Su reported a novel practical and rapid access to chiral {3[(*N-tert*-butylsulfinyl)amino]-2-oxoindolin-3-yl}acetates **33a**–**m** exhibiting a quaternary stereogenic center which was based on the zinc-mediated diastereoselective aza-Reformatsky reaction of isatin-derived chiral *N-*sulfinyl ketimines **34a**–**m** [30]. The Reformatsky reagent was in situ generated from ethyl bromoacetate (**9b**) and zinc in 2-Me-THF at 0 °C in the presence of CuCl. As shown in Scheme 13, a range of products **33a**–**m** were obtained in both moderate to high yields (65–91%) and diastereoselectivities (70–96% de). Indeed, various substituents on each carbon (C4–C7) of the indolinone ring, such as halides, alkyl, electron-withdrawing and electron-donating groups, were found compatible. In particular, the reaction of C4- and C5-substituted substrates provided uniformly high diastereoselectivies (84–96% de). The utility of this novel methodology was demonstrated by its application to the synthesis of gastrin/cholecyctokinin-B receptor antagonist AG-041R starting from Reformatsky chiral product **33a**.

**Scheme 14:**
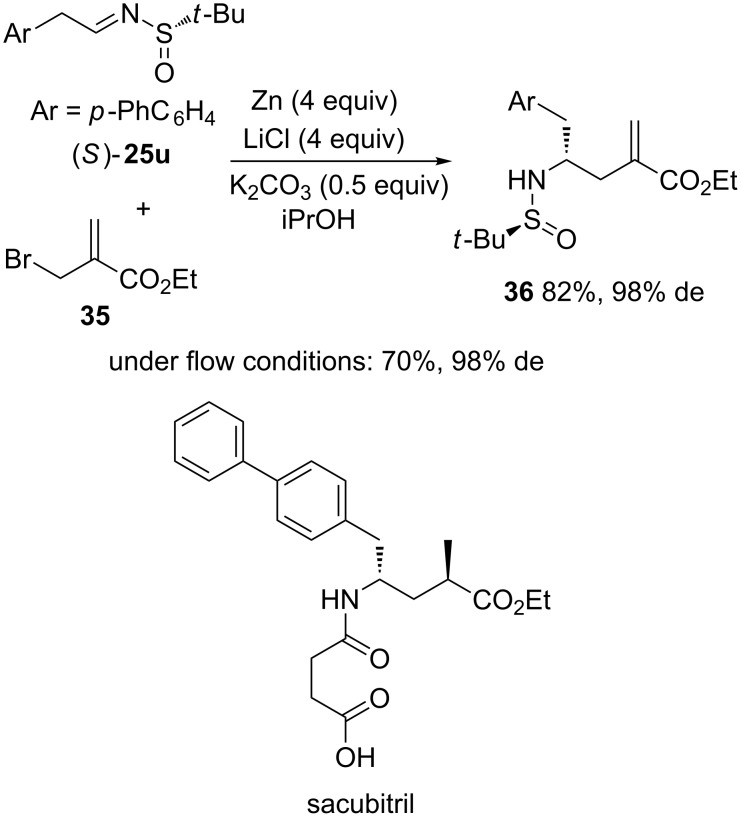
Synthesis of a precursor of sacubitril through a Zn-mediated aza-Reformatsky reaction [31].

Earlier in 2015, Ley et al. reported an efficient synthesis of a precursor of the neprilysin inhibitor sacubitril based on a zinc-mediated aza-Reformatsky-type reaction [31]. Indeed, this convergent synthesis featured a diastereoselective aza-Reformatsky-type carbethoxyallylation of (*S*)-*N*-*tert-*butylsulfinyl imine **25u** with bromide **35** performed in isopropanol as solvent in the presence of zinc, LiCl and K_2_CO_3_, which afforded the corresponding chiral acrylic ester **36** as single diastereomer (98% de) in 82% yield (Scheme 14). However, an inefficient mixing of the heterogeneous reaction media led to uncontrolled exothermic reactions on large scale, rending the process unreproducible on scale. To solve this problem, the authors developed a flow protocol using a column of activated zinc. The process involved that a reaction solution containing the two substrates with LiCl was pumped through the zinc column followed by a scavenger cartridge containing QuadraPure polymer-supported sulfonic acid and polymer-supported thiourea. This allowed diastereopure product **36** (98% de) to be obtained in 70% yield.

#### Intramolecular Reformatsky reactions

**Scheme 15:**
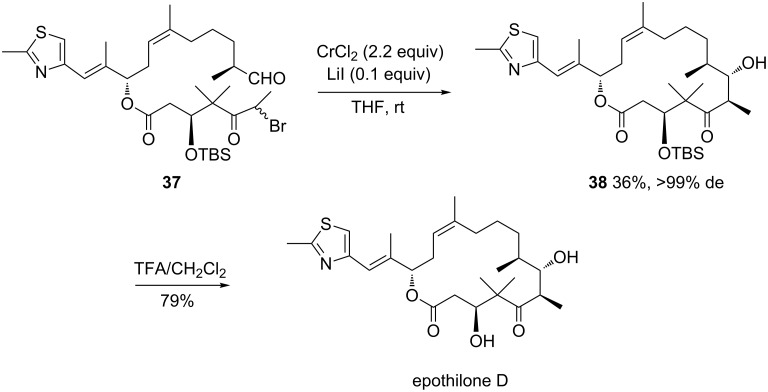
Synthesis of epothilone D through a Cr(II)-mediated Reformatsky reaction. TFA = trifluoroacetic acid [32].

In addition to diastereoselective intermolecular versions, highly efficient asymmetric intramolecular Reformatsky reactions have been developed, demonstrating the great versatility of this reaction. As a recent example, Wessjohann et al. have reported a Cr(II)-mediated Reformatsky reaction of the linear epothilone precursor **37** [32]. As shown in Scheme 15, the macrocyclization of the latter into the corresponding enantiopure 16-membered highly functionalized chiral lactone **38** (>99% de) was achieved in moderate yield (36%) through a Reformatsky reaction mediated by CrCl_2_ in THF at room temperature in the presence of LiI. A subsequently deprotection of this product by treatment with TFA led to the expected naturally occurring cytotoxic macrolide epothilone D. It must be noted that this novel highly convergent and stereocontrolled total synthesis of epothilone D clearly differed from other previously reported syntheses by the fact that it included for the first time as key step a totally diastereoselective chromium-mediated macrolactonization.

**Scheme 16:**
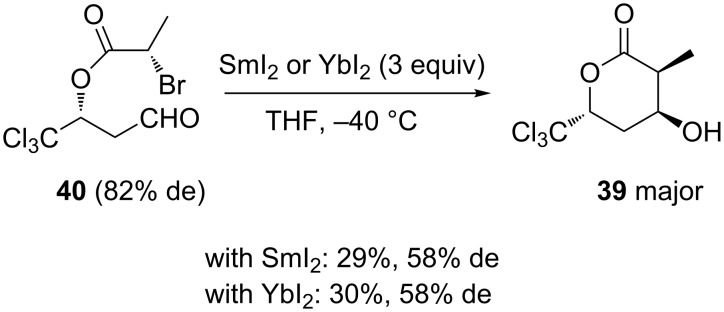
Synthesis of β-hydroxy-α-methyl-δ-trichloromethyl-δ-valerolactone through a Sm(II)- or Yb(II)-mediated Reformatsky reaction [33].

The same year, Schulze et al. reported the synthesis of chiral β-hydroxy-α-methyl-δ-trichloromethyl-δ-valerolactone (**39**) through a diastereoselective Sm(II)- or Yb(II)-mediated Reformatsky reaction of the corresponding aldehyde **40** [33]. Starting from a diastereomerically enriched aldehyde **40** (82% de), the reaction performed in THF at −40 °C in the presence of 3 equivalents of SmI_2_ or YbI_2_ provided the corresponding chiral lactone **39** as major diastereomer (58% de) in moderate yield (29 and 30%, respectively), as shown in Scheme 16. It must be noted that this study represented the first use of ytterbium to mediate a Reformatsky reaction.

**Scheme 17:**
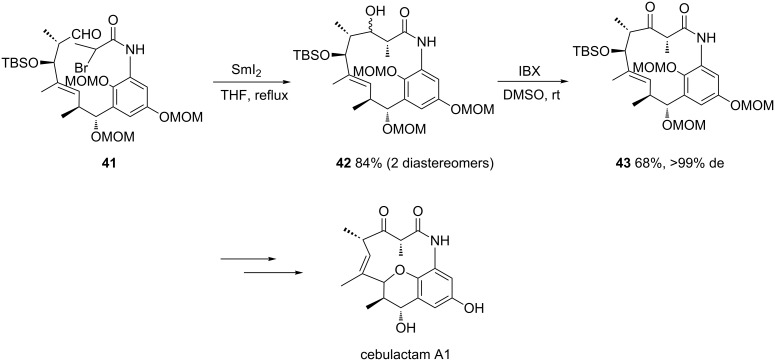
Synthesis of cebulactam A1 through a Sm(II)-mediated Reformatsky reaction. MOM = methoxymethyl [34].

In 2014, a diastereoselective SmI_2_-mediated cyclization was used as a key step by Yang and Chen in the first total synthesis of naturally occurring 11-membered macrocyclic lactam cebulactam A1 [34]. In the presence of SmI_2_ in THF at reflux, chiral amide **41** underwent an intramolecular Reformatsky reaction to give the corresponding alcohol **42** in 84% yield as a couple of diastereomers (Scheme 17). The latter diastereomeric mixture was further oxidized by treatment with IBX (2-iodoxybenzoic acid) to afford the corresponding chiral ketone **43** as a single diastereomer in 68% yield. Three supplementary steps allowed expected cebulactam A1 to be obtained in 33% yield.

**Scheme 18:**
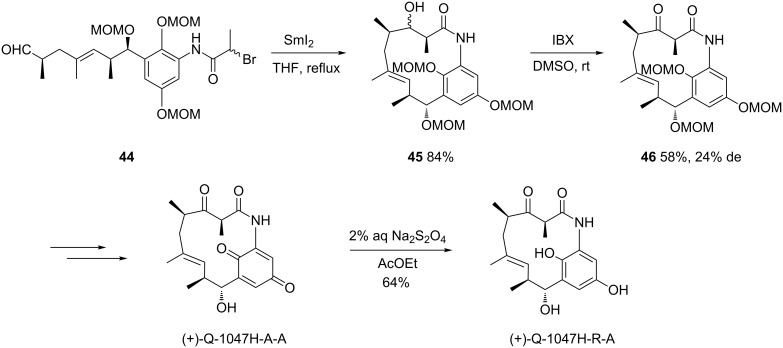
Synthesis of ansamacrolactams (+)-Q-1047H-A-A and (+)-Q-1047H-R-A through a Sm(II)-mediated Reformatsky reaction [35].

The same authors have also applied a related samarium methodology to develop the first total synthesis of chiral 13-membered lactams, such as ansamacrolactams (+)-Q-1047H-A-A and (+)-Q-1047H-R-A [35]. In this case, the intramolecular Sm(II)-mediated Reformatsky reaction was applied to chiral aldehyde **44**, leading to the corresponding alcohol **45** in 84% yield as a mixture of diastereomers (Scheme 18). The latter was subsequently oxidized by treatment with IBX to give ketone **46** as a mixture of two diastereomers (24% de) in 58% yield. After separation, the minor diastereomer was successively converted into (+)-Q-1047H-A-A and (+)-Q-1047H-R-A, allowing the reassignment of the relative stereochemistries and absolute configurations of their natural counterparts (−)-Q-1047H-A-A and (−)-Q-1047H-R-A.

### Catalytic enantioselective (aza)-Reformatsky reactions

#### Reformatsky reactions

**Scheme 19:**
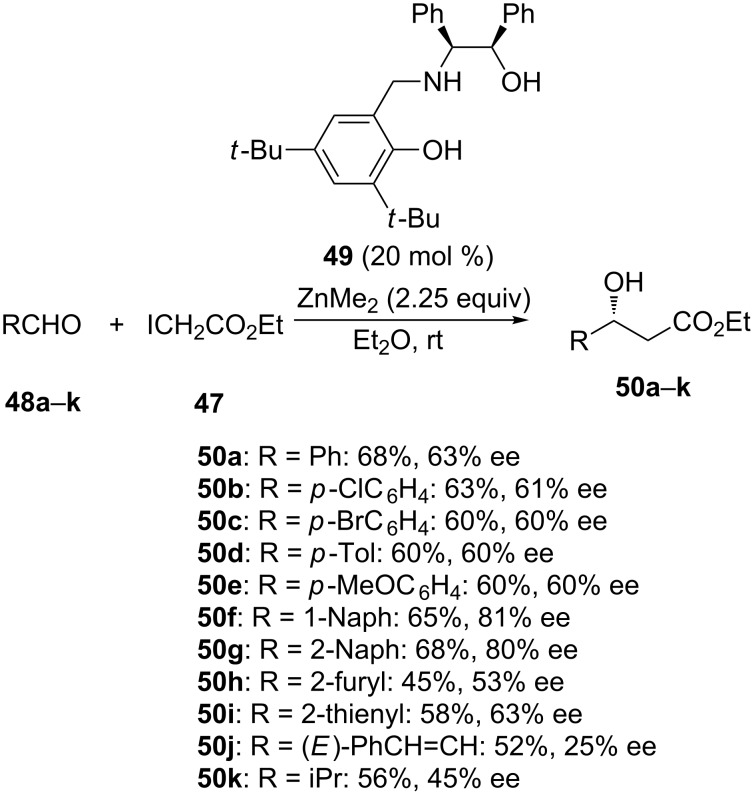
Reformatsky reaction of aldehydes with ethyl iodoacetate in the presence of a chiral 1,2-amino alcohol ligand [43].

In recent years, novel chiral ligands have been designed to promote catalytic enantioselective Reformatsky reactions [5–6,8]. In spite of this, only a few examples of highly efficient enantioselective reactions using only catalytic amounts of chiral ligands have been described so far. The first efficient examples were reported by Cozzi in 2006, dealing with ZnMe_2_-mediated reactions of ketones with ethyl iodoacetate performed with enantioselectivities of up to 86% ee in the presence of a chiral manganese salen complex [36]. The same year, this author also described the first catalytic enantioselective aza-Reformatsky reaction between in situ generated imines and ethyl iodoacetate [37]. In this case, the ZnMe_2_-mediated reaction employed *N*-methylephedrine as chiral ligand which provided the corresponding chiral β-amino esters with enantioselectivities of up to 94% ee. Later in 2008, Feringa introduced the first catalytic enantioselective version of the Reformatsky reaction involving aldehydes as electrophiles [38]. Enantioselectivities of up to 84% ee were achieved by using a BINOL derivative as chiral ligand. Ever since, other types of chiral ligands including chiral Schiff bases [39], bisoxazolidines [40], 1,2-amino alcohols [41], indolinylmethanols [42], and diarylprolinols have allowed excellent enantioselectivities to be achieved especially in the area of aza-Reformatsky reactions. Good results have also been recently described in enantioselective catalytic Reformatsky reactions performed in the presence of various chiral 1,2-amino alcohols. For example in 2014, He and Li reported the synthesis of novel chiral tridentate ligands from the reaction between (1*R*,2*S*)-2-amino-1,2-diphenylethanol and substituted salicylaldehydes [43]. These chiral 1,2-amino alcohols were further investigated as ligands in the enantioselective Reformatsky reaction of aldehydes with ethyl iodoacetate (**47**), leading to select ligand **49** as optimal one. As shown in Scheme 19, the reaction of a range of aldehydes **48a**–**k** performed at room temperature in diethyl ether in the presence of 20 mol % of this ligand and 2.25 equivalents of ZnMe_2_ provided the corresponding chiral β-hydroxy ethyl esters **50a**–**k** in moderate yields (52–68%) and low to good enantioselectivities (25–81% ee). The best enantioselectivities (80–81% ee) were achieved in the reaction of 1-naphthaldehyde and 2-naphthaldehyde while acyclic α,β-unsaturated aldehydes and aliphatic aldehydes gave the lowest enantioselectivities (25–45% ee). This study represented the first use of chiral 1,2-amino alcohols as tridentate ligands for asymmetric Reformatsky reaction of aldehydes.

**Scheme 20:**
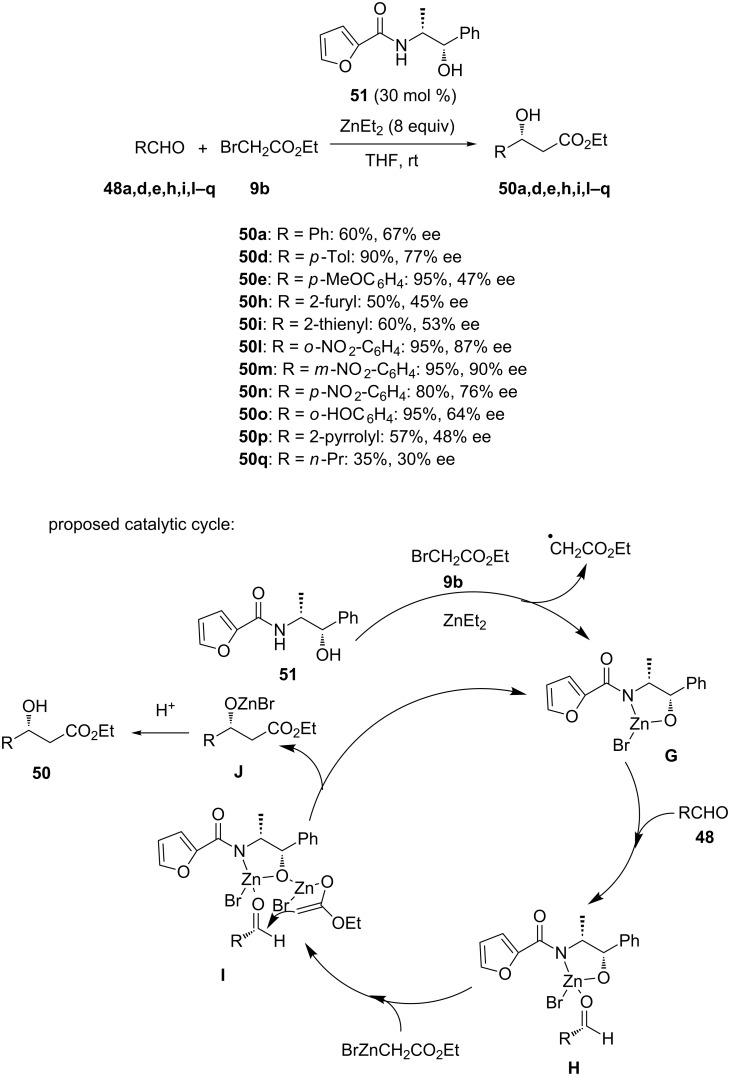
Reformatsky reaction of aldehydes with ethyl bromoacetate in the presence of a chiral amide ligand [44].

The zinc-mediated Reformatsky reaction of aldehydes with ethyl bromoacetate (**9b**) was investigated by Velmathi and Ananthi in the presence of a chiral amide ligand derived from (+)-norephedrine and 2-furoic acid [44]. Indeed, using 30 mol % of chiral amide ligand **51** in THF at room temperature, the reaction of a range of aldehydes **48a,d,e,h,i,l**–**q** with ethyl bromoacetate (**9b**) led to the corresponding chiral β-amino alcohols **50a,d,e,h,i,l**–**q** in moderate to excellent yields (35–95%) and enantioselectivities (30–90% ee), as shown in Scheme 20. Aromatic aldehydes generally produced the corresponding products in better yields (50–95% vs 35%) and enantioselectivities (45–90% ee vs 33% ee) than aliphatic aldehydes. The authors demonstrated that the presence of air in this process was essential for the effective C−C bond formation. They proposed the catalytic cycle depicted in Scheme 20 which begins with the reaction between chiral ligand **51**, ZnEt_2_ and ethyl bromoacetate (**9b**) to give complex **G**. Addition of the aldehyde to the latter results in the formation of complex **H**. A subsequent addition of another molecule of BrZnCH_2_CO_2_Et to this complex results in the formation of complex **I**. In complex **I**, the double bond of the zinc enolate attacks the carbonyl group of the aldehyde providing compound **J** which led to the final product **50** under acidic work-up conditions. The formation of complex **G** was demonstrated through IR spectroscopy. Indeed, the frequency of the NH and OH groups in this complex was higher than that in the single ligand **51**. On the other hand, the frequency of the carbonyl group was the same as that of pure ligand **51**, showing that this ligand coordinated to zinc metal through its OH and NH groups.

**Scheme 21:**
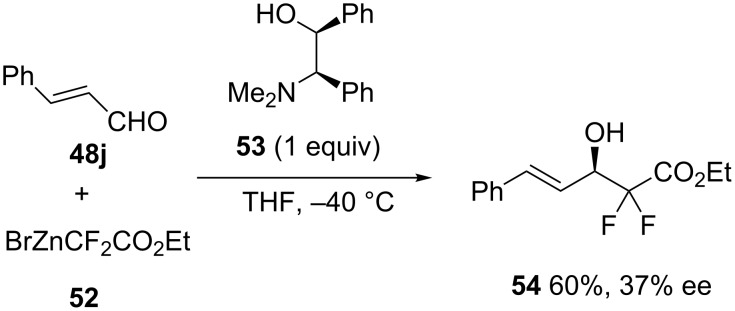
Reformatsky reaction of cinnamaldehyde with ethyl bromozinc-α,α-difluoroacetate in the presence of a stoichiometric amount of a chiral 1,2-amino alcohol ligand [45].

The enantioselective Reformatsky reaction between cinnamaldehyde (**48j**) and ethyl bromozinc-α,α-difluoroacetate (**52**) was recently investigated by Wu et al. [45]. Among a range of different types of chiral ligands investigated, including various 1,2-amino alcohols and BINOL (1,1’-bi-2-naphthol) derivatives, ligand **53** was selected as optimal ligand when used at a stoichiometric amount in THF at −40 °C. As shown in Scheme 21, the corresponding fluorinated chiral β-amino alcohol **54** was formed in both moderate yield (60%) and enantioselectivity (37% ee).

**Scheme 22:**
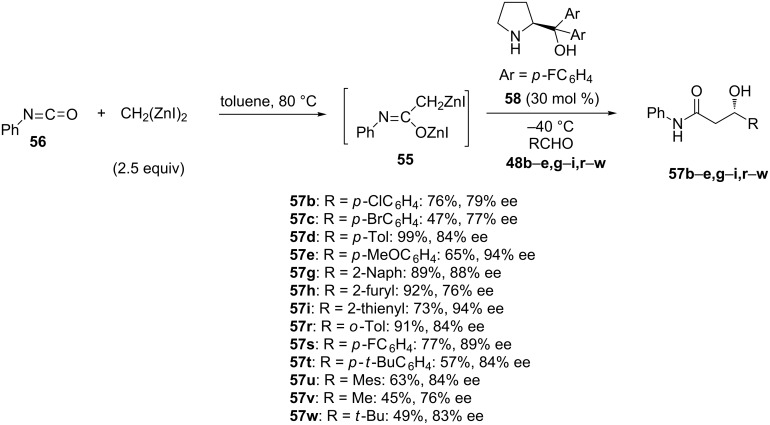
Reformatsky reaction of aldehydes with an enolate equivalent prepared from phenyl isocyanate and CH_2_(ZnI)_2_ performed in the presence of a chiral diarylprolinol ligand [46].

Finally, good to excellent enantioselectivities (76–94% ee) combined to moderate to quantitative yields (45–99%) were reported by Matsubara and Haraguchi in the enantioselective Reformatsky reaction of aldehydes **48b**–**e,g**–**i,r**–**w** with enolate equivalent **55** prepared from phenyl isocyanate **56** and CH_2_(ZnI)_2_ in toluene at 80 °C [46]. As shown in Scheme 22, the latter reacted at −40 °C with a range of (hetero)aromatic as well as aliphatic aldehydes **48b**–**e,g**–**i,r**–**w** to give the corresponding chiral amides **57b**–**e,g**–**i,r**–**w**. The lowest yields (45–49%) and enantioselectivities (76–83% ee) were obtained in the reaction of aliphatic aldehydes whereas good to excellent enantioselectivities (76–94% ee) combined to moderate to quantitative yields (47–99%) were achieved in the reaction of (hetero)aromatic aldehydes. These results were achieved by using 30 mol % of diarylprolinol **58** selected as optimal chiral ligand among a range of related molecules.

#### Aza-Reformatsky reactions

**Scheme 23:**
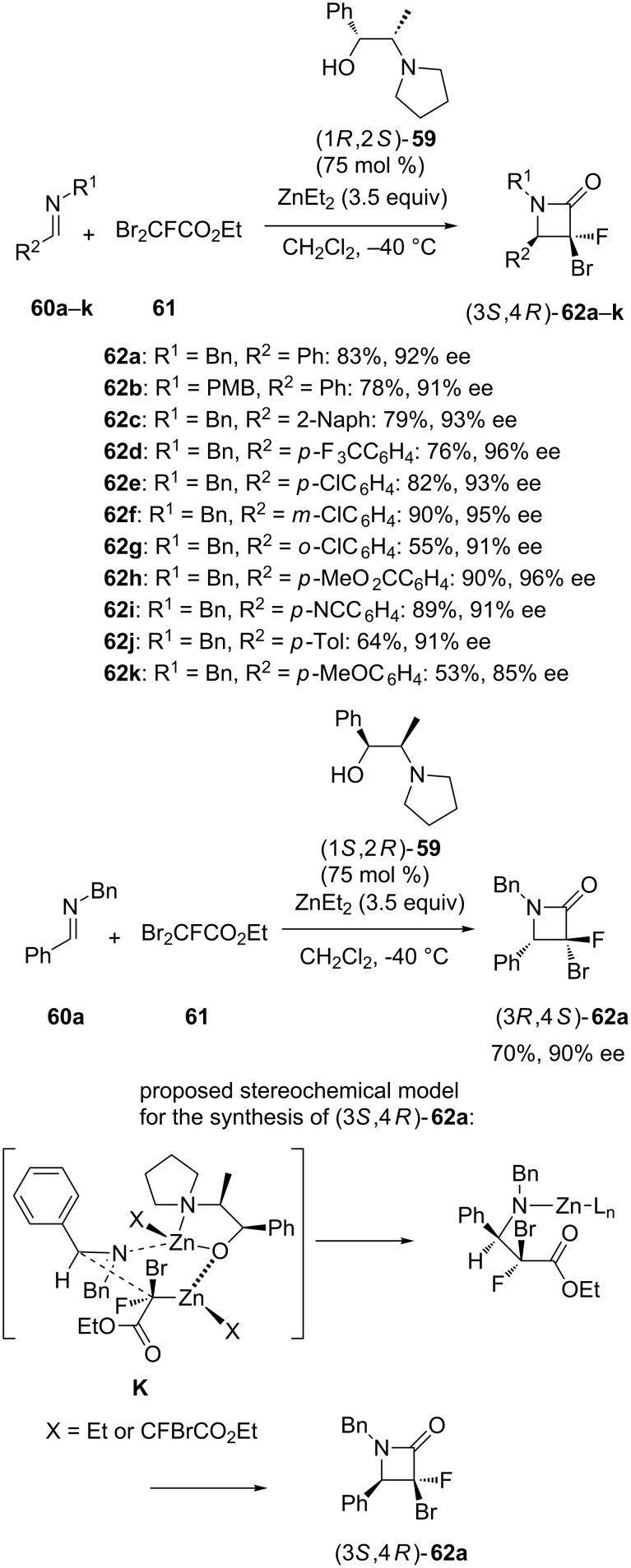
Domino aza-Reformatsky/cyclization reactions of imines with ethyl dibromofluoroacetate in the presence of chiral 1,2-amino alcohol ligands [47].

Especially, several excellent results have been recently reported in the area of enantioselective catalytic aza-Reformatsky reactions by using chiral 1,2-amino alcohols and diarylprolinols as ligands. For example, chiral 1,2-amino alcohol (1*R*,2*S*)-**59** was found as a highly efficient ligand employed at 75 mol % of catalyst loading in the enantioselective ZnEt_2_-mediated aza-Reformatsky reaction of aromatic imines **60a**–**k** with ethyl dibromofluoroacetate (**61**) in dichloromethane at −40 °C [47]. Actually, the corresponding Reformatsky acyclic products cyclized under the reaction conditions to give directly the final chiral α-bromo-α-fluoro-β-lactams (3*S*,4*R*)-**62a**–**k** in moderate to high yields (53–90%) and high enantioselectivities (85–96% ee), as shown in Scheme 23. This method represented the first ligand-promoted aza-Reformatsky approach using a halofluoroacetate. When the opposite enantiomer of the ligand (1*S,*2*R*)-**59** was used under the same reaction conditions, it provided the corresponding (3*R*,4*S*) product **62a** in 70% yield and 90% ee starting from the corresponding imine **60a** (Scheme 23). Unfortunately, the scope of this domino Reformatsky/cyclization reaction could not be extended to aliphatic imines since they led to complex mixtures. To explain the stereoselectivity of the reaction providing (3*S*,4*R*)-**62a**, the authors proposed transition state **K** arisen from coordination between zinc ions and imine nitrogen, ligand **59**, and the Reformatsky reagent (Scheme 23).

**Scheme 24:**
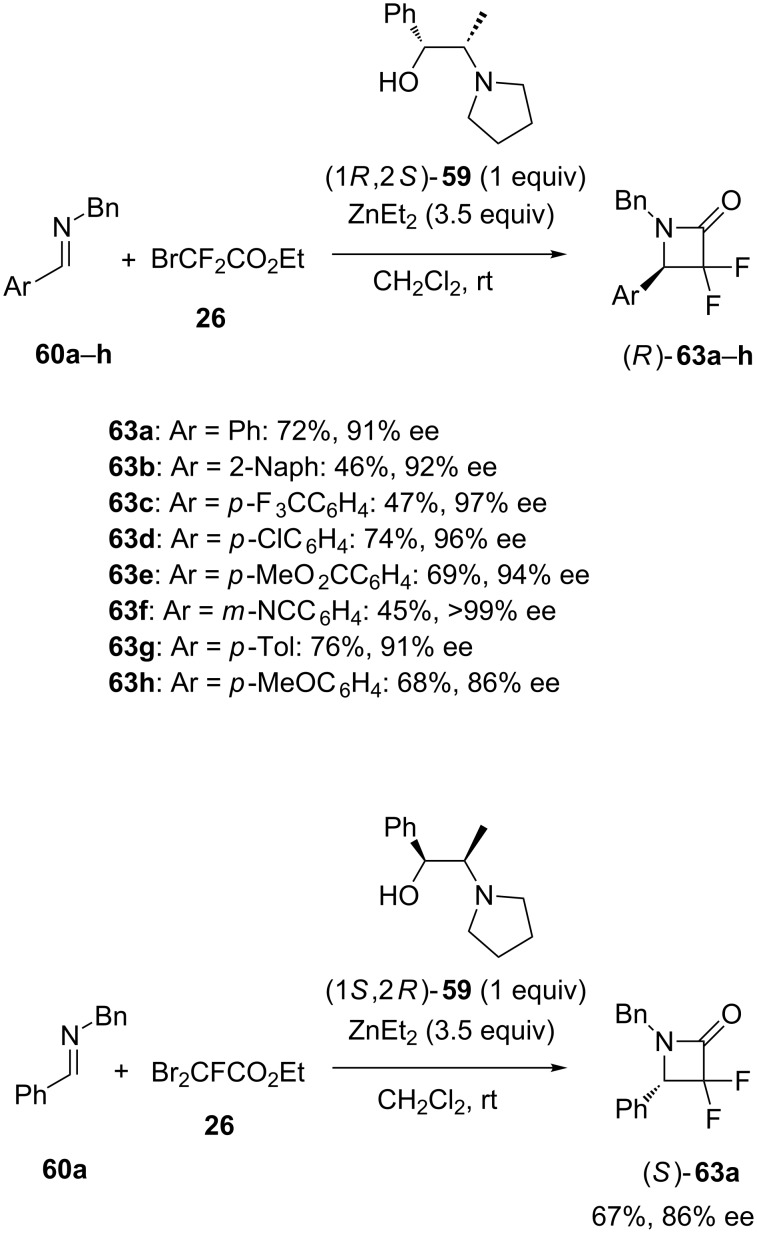
Domino aza-Reformatsky/cyclization reactions of imines with ethyl bromodifluoroacetate in the presence of a stoichiometric amount of chiral 1,2-amino alcohol ligands [48].

As an extension of the precedent methodology, the same ligand (1*R*,2*S*)-**59** was applied to promote the enantioselective domino aza-Reformatsky/cyclization reaction of aromatic imines **60a**–**h** with ethyl bromodifluoroacetate (**26**) [48]. In this case, a stoichiometric amount of this ligand was required to achieve at room temperature the corresponding chiral α,α-difluoro-β-lactams (*R*)-**63a**–**h** in moderate to good yields (45–76%) and high to excellent enantioselectivities (86–99% ee), as shown in Scheme 24. Using enantiomeric ligand (1*S*,2*R*)-**59** under the same reaction conditions allowed the opposite enantiomer (*S*)-**63a** to be achieved in 67% yield and 86% ee starting from the corresponding imine **60a** (Scheme 24). This convenient methodology constituted a novel and simple approach to chiral fluorinated building blocks.

**Scheme 25:**
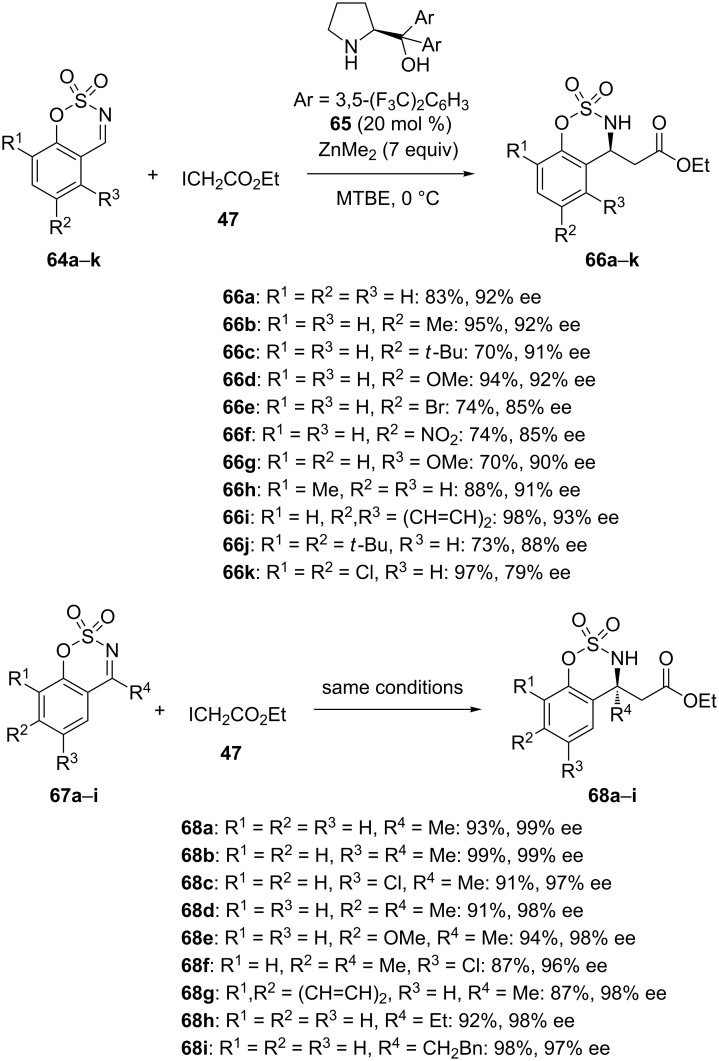
Aza-Reformatsky reactions of cyclic imines with ethyl iodoacetate in the presence of a chiral diarylprolinol ligand [49].

In 2016, Pedro and Vila envisioned the first use of cyclic imines, such as benzoxathiazine 2,2-dioxides, as electrophiles in the catalytic enantioselective aza-Reformatky reaction [49]. Indeed, these compounds constitute good partners for asymmetric catalysis since they exhibit a rigid structure reducing the conformational mobility and avoiding the *E/Z* isomerization, thus facilitating the stereodifferentiation. Investigating several types of chiral ligands, including 1,2-amino alcohols, quinine, and diarylprolinols, in the aza-Reformatsky reaction of variously substituted aromatic aldimines **64a**–**k** with ethyl iodoacetate (**47**), chiral diarylprolinol **65** was selected as optimal ligand. As shown in Scheme 25, when this reaction was performed at 0 °C with only 20 mol % of this ligand combined to 7 equivalents of ZnMe_2_ in MTBE (methyl *tert*-butyl ether) as solvent, it afforded the corresponding β-amino esters **66a–k** in high to quantitative yields (70–98%) and high enantioselectivities (79–93% ee). Notably, even sterically hindered cyclic imines bearing two substituents were compatible, providing the corresponding products in high enantioselectivities (79–93% ee). Moreover, the use of ketimines in such reactions represented a challenge, owing to their low reactivity and steric bulkiness. Despite this, these authors could extend their reaction conditions to this type of electrophiles for the first time, as illustrated in Scheme 25. Therefore, a range of aromatic ketimines **67a–i** reacted with ethyl iodoacetate (**47**) to give the corresponding chiral β-amino esters **68a–i** exhibiting a quaternary stereocenter in uniformly excellent yields (87–99%) and enantioselectivities (96–99% ee).

**Scheme 26:**
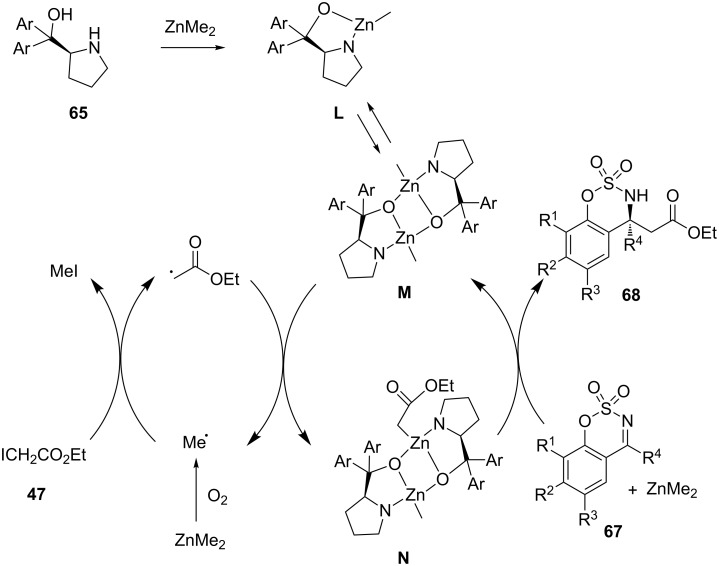
Mechanism for aza-Reformatsky reaction of cyclic imines with ethyl iodoacetate in the presence of a chiral diarylprolinol ligand [49].

To explain these results, the authors have proposed the catalytic cycle depicted in Scheme 26 in which ligand **65** was deprotonated by ZnMe_2_ to generate complex **L** in equilibrium with dimeric complex **M**. Then, the addition of ethyl iodoacetate (**47**) was accelerated in the presence of ZnMe_2_ and oxygen through a cycle in which ZnMe_2_ acted as a source of methyl radicals which reacted with ethyl iodoacetate giving ethyl acetate radical. The latter added to complex **M** to give complex **N**. Then complex **N** assisted the nucleophilic addition of ethyl iodoacetate to the *Si* face of the cyclic imine **67** affording the final β-amino ester **68** and regenerating dimer **M**.

**Scheme 27:**
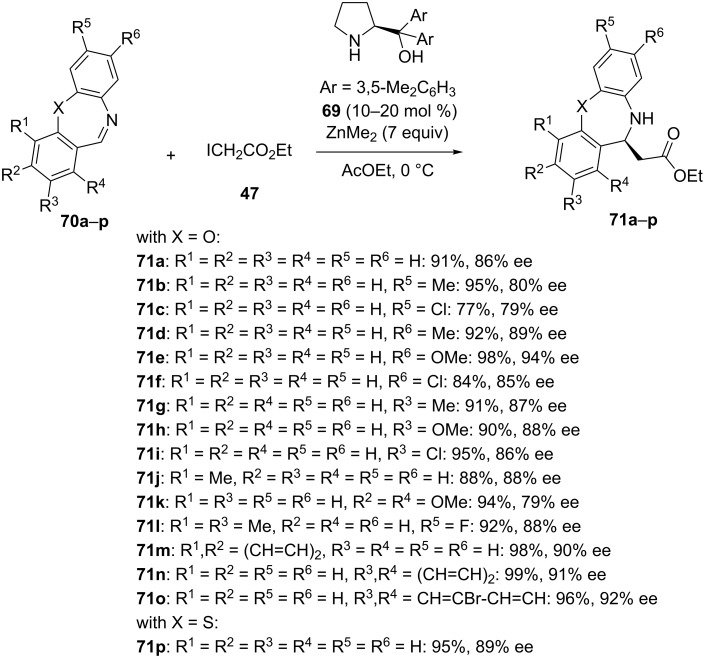
Aza-Reformatsky reaction of dibenzo[*b,f*][1,4]oxazepines and dibenzo[*b,f*][1,4]thiazepine with ethyl iodoacetate in the presence of a chiral diarylprolinol ligand [50].

In 2017, a related chiral ligand **69** employed at only 10–20 mol % of catalyst loading was reported by the same authors to be capable of catalyzing in combination with ZnMe_2_ in ethyl acetate as solvent at 0 °C the enantioselective aza-Reformatsky reaction of seven-membered cyclic imines, such as dibenzo[*b*,*f*][1,4]oxazepines (X = O) and dibenzo[*b*,*f*][1,4]thiazepine (X = S) **70a–p** [50]. As shown in Scheme 27, the reaction of these electrophiles with ethyl iodoacetate (**47**) led to the corresponding chiral β-amino esters **71a–p** with both high yields (77–99%) and enantioselectivities (79–94% ee). A range of both electron-donating and electron-withdrawing substituents at different positions of the two aromatic rings were compatible.

## Conclusion

This review demonstrates that much progress has been achieved in the last five years in the field of asymmetric (aza)-Reformatsky reactions, involving both chiral reagents and chiral ligands. These significant advances have made the Reformatsky methodology more readily applicable than before for modern organic synthesis. For example, as illustrated in the first part of the review, a number of total syntheses of natural and/or biologically active products included as key steps highly diastereoselective (aza)-Reformatsky-type inter- as well as intramolecular reactions based on the use of chiral substrates generally performed under mild and neutral conditions. Among them, many syntheses of naturally occurring products have been described for the first time, such as those of the glucose-regulated protein 78 expression inhibitor agent prunustatin A, the antiproliferative agent apratoxin E and its C30 epimer, prebiscibactin, cebulactam A1, and ansamacrolactams. In addition, novel total syntheses of prostaglandin E_2_, the cytotoxic agent epothilone D, and protein inhibitor ABT-737 along with formal syntheses of the protein inhibitor tedanolide C, the neprilysin inhibitor sacubitril, the antiproliferative agent jatrophane diterpene Pl-3, and gastrin/cholecyctokinin-B receptor antagonist AG-041R have been recently achieved on the basis of highly diastereoselective (aza)-Reformatsky reactions mediated by Zn, SmI_2_, SnCl_2_, or CrCl_2_. Besides these total syntheses of important products, novel diastereoselective procedures have also been described using chiral reagents. For example, the first efficient synthesis of chiral β-trifluoromethyl β-amino acid derivatives containing a quaternary stereocenter adjacent to the amine function has been reported with up to >98% de through diastereoselective Zn-mediated aza-Reformatsky reactions between chiral α-trifluoromethyl *N-tert-*butylsulfinyl hemiaminals and α-bromoesters. In the same area, diastereoselectivities of up to >90% de were achieved in the Zn-mediated aza-Reformatsky reactions of chiral sulfinyl imines with fluorobromoesters/ketones. Moreover, isatin-derived chiral *N-*sulfinyl ketimines were found effective electrophiles in the Zn-mediated aza-Reformatsky reaction with ethyl bromoacetate since the corresponding chiral products were obtained with diastereoselectivities of up to 96% de. The second part of the review shows that the even more interesting field of catalytic enantioselective (aza)-versions of the Reformatsky reaction is fast-growing with the possibility of performing these reactions under homogeneous conditions. For example, excellent enantioselectivities of up to 96% ee were recently reported in the first use of halofluoroacetates as precursors of Reformatsky reagents in enantioselective reactions with imines catalyzed by catalytic amounts of chiral 1,2-amino alcohol ligands in the presence of ZnEt_2_ as the zinc source. Moreover, the first use of cyclic imines, including low reactive cyclic ketimines, in enantioselective aza-Reformatsky reactions with ethyl iodoacetate was described in 2016 with remarkable enantioselectivities (up to 99% ee) achieved by using 20 mol % of another chiral 1,2-amino alcohol ligand in the presence of ZnMe_2_ as the zinc source. In 2017, the use of 10–20 mol % of another type of chiral ligand, such as a diarylprolinol, allowed the first enantioselective catalytic aza-Reformatsky reaction of dibenzo[*b*,*f*][1,4]oxazepines with ethyl iodoacetate with enantioselectivities of up to 94% ee performed in the presence of ZnMe_2_. In spite of the discovery of many effective chiral ligands to promote the catalytic enantioselective Reformatsky reaction in the last few years, further efforts are required to improve limited substrate scopes, yields, and enantioselectivities. In the future, the involvement of other types of ligands will have to be investigated in these reactions with better understanding of the transition states. More generally, only limited studies have been undertaken so far to replace organic solvents by aqueous media to render asymmetric Reformatsky reactions greener.
